# Transparent masks reduce the negative impact of opaque masks on understanding emotional states but not on sharing them

**DOI:** 10.1186/s41235-022-00411-8

**Published:** 2022-07-07

**Authors:** Sarah D. McCrackin, Sabrina Provencher, Ethan Mendell, Jelena Ristic

**Affiliations:** Department of Psychology, McGill University, 2001 McGill College Avenue, Montreal, QC H3A 1G1 Canada

**Keywords:** Lower face occlusion, Facial features, Affective empathy, Affective theory of mind, Face masks, Transparent masks

## Abstract

While face masks provide necessary protection against disease spread, they occlude the lower face parts (chin, mouth, nose) and consequently impair the ability to accurately perceive facial emotions. Here we examined how wearing face masks impacted making inferences about emotional states of others (i.e., affective theory of mind; Experiment 1) and sharing of emotions with others (i.e., affective empathy; Experiment 2). We also investigated whether wearing transparent masks ameliorated the occlusion impact of opaque masks. Participants viewed emotional faces presented within matching positive (happy), negative (sad), or neutral contexts. The faces wore opaque masks, transparent masks, or no masks. In Experiment 1, participants rated the protagonists’ emotional valence and intensity. In Experiment 2, they indicated their empathy for the protagonist and the valence of their emotion. Wearing opaque masks impacted both affective theory of mind and affective empathy ratings. Compared to no masks, wearing opaque masks resulted in assumptions that the protagonist was feeling less intense and more neutral emotions. Wearing opaque masks also reduced positive empathy for the protagonist and resulted in more neutral shared valence ratings. Wearing transparent masks restored the affective theory of mind ratings but did not restore empathy ratings. Thus, wearing face masks impairs nonverbal social communication, with transparent masks able to restore some of the negative effects brought about by opaque masks. Implications for the theoretical understanding of socioemotional processing as well as for educational and professional settings are discussed.

Face masks remain one of the first lines of defense against virus spread (Eikenberry et al., 2020; Leung et al., 2020; Prather et al., 2020). However, since masks occlude the lower parts of the face, they also obscure important facial cues needed for social communication (Mheidly et al., 2020), such as face identity (Freud et al., 2020; Noyes et al., 2021) and emotion (McCrackin et al., 2022; Carbon, 2020; Grundmann, Epstude & Scheibe, 2020; Noyes et al., 2021). Thus, the inability to accurately perceive facial emotions due to facial occlusion may be an important contributing factor in downstream processes related to complex emotion recognition. In this study we examined how wearing face masks impacted relating to others by measuring participants’ ability to infer the mask wearer’s emotional states or to engage in affective theory of mind (Experiment 1) and to share those emotional states with them or engage in affective empathy (Experiment 2). That is, going beyond simply recognizing facial emotions, here we examined how facial occlusion by masks affected the ability to infer and share a masked protagonist’s emotional states.

Affective theory of mind is a socioemotional ability that is intimately tied with emotion recognition (Decety et al., 2015; Stewart et al. 2019). While basic emotion recognition requires identifying physical facial features that match an emotion (e.g., a smile reflects happiness; Decety et al., 2015; Stewart et al. 2019), affective theory of mind enables making a more complex inference about an individual’s emotional state (e.g., He/she is sad; Decety et al., 2015; Stewart et al. 2019). The emotional attribution typically involves incorporating knowledge from multiple sources to determine what another individual is feeling (Decety et al., 2015; Stewart et al. 2019; Baron-Cohen & Cross, 1992; Clark et al., 2008; Mier et al., 2010) and critically beyond perception of the individual’s facial cues, includes a key additional consideration of the person’s mind, desires, and feelings (Decety et al., 2015; Stewart et al. 2019). Unsurprisingly, individuals who are better at inferring the emotional states of others demonstrate higher social competence (Bosacki & Wilde Astington, 1999), initiate prosocial behavior more frequently (Imuta et al., 2016), and benefit from improved psychosocial functioning (Nader-Grosbois & Day, 2011). As facial occlusion by face masks has been shown to alter basic emotion recognition significantly (McCrackin et al., 2022; Carbon, 2020; Grundmann, Epstude & Scheibe, 2020; Noyes et al., 2021), it is likely that facial occlusion may also exert a similar impact on emotional inferences involved in affective theory of mind.

Affective empathy is related to affective theory of mind (Decety, Lewis & Cowell, 2015; de Vignemont & Singer, 2006; Lieberman, 2007; Kanske et al. 2015) but distinct in that on top of emotional understanding, it also involves emotional contagion, where one’s own emotions become altered through shared emotions with others. For example, one might experience negative empathy by feeling sad when their friend is upset, or positive empathy by feeling happy when their friend is happy. Both positive and negative empathy have been associated with increased social competence (Allemand et al., 2015) and prosociality (Telle & Pfister, 2016). Affective empathy is related to emotion recognition and affective theory of mind because it requires perceiving and inferring an emotional state as a precursor to emotional sharing. This suggests that mask-related impairments in emotion recognition and understanding may also have effects on emotional sharing and affective empathy processes.

Experiment 1 investigated the impact of lower facial occlusion by face masks on affective theory of mind. Experiment 2 investigated the impact of lower face facial occlusion by face masks on affective empathy. Both experiments also examined whether face covering by clear masks, which allows for visual perception of faces, would ameliorate the impact of facial occlusion by opaque masks on emotional understanding and sharing. We reasoned that any impact of visual occlusion by masks would stem from reduced ability to perceive facial expressions, as demonstrated by previous work on face masks and emotion recognition (McCrackin et al., 2022; Carbon, 2020; Grundmann, Epstude & Scheibe, 2020; Noyes et al., 2021). Transparent masks have recently emerged as an effective protection option which would also allow for the visual transmission of lower face features, suggesting a relatively straightforward way for preserving the protective efficacy of masks while at the same time reducing the socioemotional communicative barriers associated with facial occlusion (e.g., McCrackin et al., 2022; Mheidly et al., 2020). We modeled the opaque mask appearance after a common surgical mask, and the transparent mask design after an FDA approved clear mask.

## Experiment 1

Experiment 1 investigated whether occluding face parts with an opaque, clear, or no mask impacted the ability to make inferences about the mask wearer’s emotional state, or so-called affective theory of mind (Decety et al. 2015; de Vignemont & Singer, 2006; Kanske et al. 2015; Lieberman, 2007).

One way in which face masks may alter affective theory of mind is by blocking the visual transmission of lower face cues which are critical for perceiving facial emotions (McCrackin et al., 2022; Carbon, 2020; Grundmann, Epstude & Scheibe, 2020; Noyes et al., 2021). While affective theory of mind is distinct from basic emotion recognition (Decety et al., 2015; Stewart et al. 2019), the two abilities are highly related. The accurate recognition of emotional expressions is commonly considered as a key precursor to more complex understanding of emotions in others or the affective theory of mind, which requires an integration of physical cues like facial expressions with contextual cues such as emotion understanding (Baron-Cohen & Cross, 1992; Clark et al., 2008; Mier et al., 2010). Wearing opaque face masks has been shown to significantly lower the ability to recognize facial expressions because they visually occlude lower face features (e.g., a smiling mouth or wrinkled nose; McCrackin et al., 2022; Carbon, 2020; Grundmann, Epstude & Scheibe, 2020; Noyes et al., 2021). As such, facial occlusion by opaque masks may prevent the typical perception and integration of facial expression information with larger emotional context while in contrast transparent masks may facilitate the perception of facial expressions and thus facilitate affective theory of mind.

To investigate this question, in Experiment 1 participants viewed photographs of happy, neutral, and sad faces wearing opaque masks, transparent masks, or no masks (unoccluded faces). Each photograph was presented within with a congruent positive, neutral, or negative sentence providing emotional context. The sentences described the individual in various situations, so that participants had access to both emotional information from the face and the specific context for that individual’s emotion (e.g., “Her cat was *found/fed/lost* yesterday afternoon). Participants judged emotional valence and emotion intensity for each face using a Likert scale. We reasoned that affective theory of mind would be reduced for individuals wearing opaque masks relative to those wearing no masks such that participants would believe that individuals were feeling more neutral and less intense emotions. We also reasoned that affective theory of mind ratings for faces wearing transparent face masks may resemble affective theory of mind ratings for unoccluded faces, given that transparent masks allow for the visual perception of lower face cues.

## Methods

The study was pre-registered (https://osf.io/8pa9u). Anonymized raw/summarized data are available at https://osf.io/t698c/?view_only=dacda69474074b10820ee5c1ce47aa0d.

### Participants

The final sample consisted of 123 participants (36 male, 87 female, mean age: 30.64, *SD* = 1.11; average years of education from grade one: 15.14, *SD* = 0.24).^1^ They were recruited from the Prolific Academic platform (http://prolific.co) and given monetary compensation. Our target sample was 120 participants. This goal (1) reflected an a priori power analysis determining that data from 112 participants were needed to achieve 0.90 power (α=.05) to detect a medium correlation effect size (*r* = 0.3) between self-report emotion measures and behavioral data, and (2) is consistent with the sample of 120 participants that we used in our study which investigated the impact of face masks on emotion recognition (McCrackin et al., 2022). All procedures were approved by the McGill University research ethics board.

### Apparatus and stimuli

**Fig. 1:**
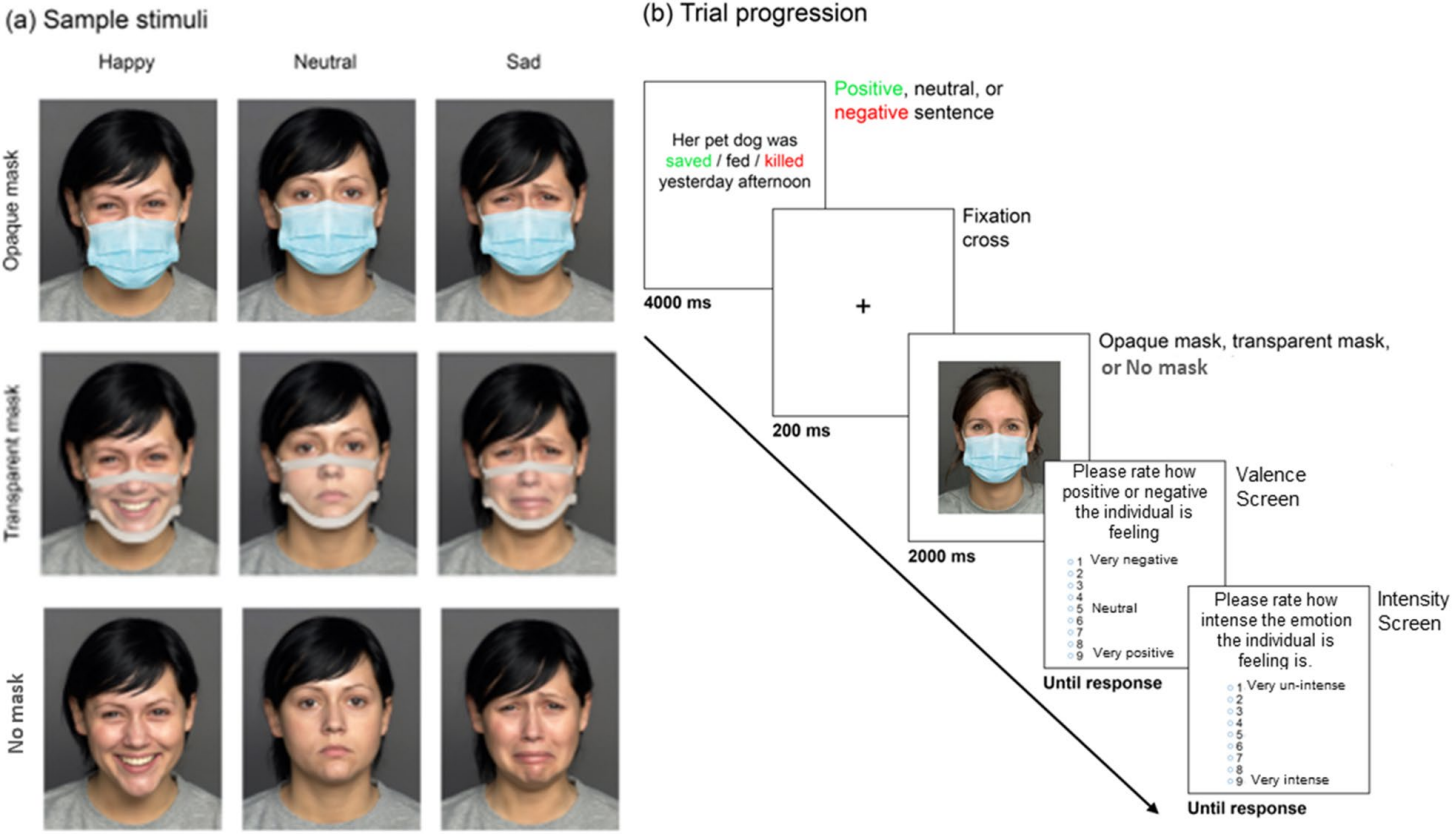
**a** Example photographs depicting happy, neutral, and sad expressions for Opaque mask, Transparent mask, and No mask conditions; **b** Sample trial progression. Participants were presented with a contextual sentence for 4000 ms. A fixation cross in duration of 200 ms preceded the presentation of a stimulus photograph displaying a congruent emotion while wearing an opaque mask, a transparent mask, or no mask. The face stimulus was shown for 2000 ms. Affective theory of mind responses were collected by asking participants to rate, on a scale from 1 (negative/low) to 9 (positive/high), how positive or negative the protagonist was feeling and the intensity of their emotion. Each response screen remained visible until response

The experiment was programmed using Testable (https://www.testable.org/). Data collection was completed online on participants’ personal computers, with stimuli sized to their screens. Face stimuli (Fig. 1) were images of sixty individuals (half female) each displaying happy, sad, and neutral expressions. Faces were sourced from the FACES (Ebner, Riediger, and Lindenbergerand, 2010; 26 males and 26 females selected^2^) and Karolinska Directed Emotional Faces (KDEF; Lundqvist et al., 1998; 4 males and 4 females selected^3^) databases. Both databases have been validated for high accuracy of emotional expressions (Ebner, Riediger, and Lindenbergerand, 2010; Goeleven et. al., 2008) with all selected identities displaying pre-validated hit rates over 0.81 averaged across the happy, sad, and neutral expressions.

A photograph of a surgical mask and a computer-generated model of a transparent mask were manipulated using Adobe Photoshop CS6 and scaled for each individual face so that the bottom of the mask reached the lower edge of the chin, the top reached the bridge of the nose, and the first outer crease reached the edges of the cheeks. The transparent mask design was based on the ClearMask™ which has been FDA approved for medical use.

Twenty-five affective sentence themes were selected from McCrackin and Itier (2021a, 2021b). Each theme had three variations referring to the protagonist experiencing positive, neutral, and negative situations (e.g., “Her cat was *found/fed/lost* yesterday afternoon”), and each has been validated to ensure that they elicit positive (positive valence condition), neutral (neutral valence condition), and negative (negative valence condition) emotional states, respectively, when they are paired with a neutral facial emotional expression. The sentences we selected were those with highest overall emotion scores (see McCrackin & Itier, 2021a, 2021b; the list of sentences used in the present study is included in Appendix 1) and did not depict any contexts that referenced illness or illness related topics.

A total of 120 sentences were used. Individual sentences were paired with 60 images referencing a male protagonist (20 neutral, 20 negative, 20 positive emotions) and 60 images referencing a female protagonist (20 neutral, 20 negative, 20 positive emotions).

### Design

The study was a repeated measures design with two factors: Mask (Opaque, Transparent, and No mask) and Emotion (Positive, Neutral, and Negative). The Mask factor manipulated opaque, transparent, and no mask conditions. Each face identity was included in each mask condition; however, this was controlled across participants such that one participant would only see one identity paired with one mask type (opaque, transparent, or no mask) to control for face familiarity within participants.

The Emotion factor manipulated positive (happy), neutral, and negative (sad) facial expressions and emotional sentence contexts. Facial expression and emotional sentences were always congruent (e.g., happy facial expressions were paired with happy context sentences). Like mask type, sentence context was also controlled across participants such that the pairing of face identities and contexts varied between the three versions.

### Procedure

An example trial sequence is illustrated in Fig. 1b. On each trial, participants first read an affective sentence describing an individual in a positive, neutral, or negative context. The sentence was shown for 4000 ms. Then a fixation cross appeared for 200 ms and was replaced by photograph of a protagonist’s face showing a happy, neutral, or negative facial expression and wearing an opaque mask, a transparent mask, or no mask. The emotional expression shown by the face was congruent with both sentence gender (i.e., male faces for male pronouns, female faces for female pronouns) and valence (i.e., happy expressions for positive sentences, neutral expressions for neutral sentences, and sad expressions for negative sentences). The face image remained visible for 2000 ms. Participants were then asked to rate (a) the assumed emotional valence of the protagonist on a 9-point Likert scale ranging from 0 (“Very negative”) to 9 (“Very positive”) and (b) the assumed emotional intensity of the protagonist on a 9-point scale ranging from 0 (“Very un-intense”) to 9 (“Very intense”).^4^

The combination of the two factors of Mask (Opaque, Transparent, and No mask) and Emotion (Positive, Neutral, and Negative) yielded 9 experimental conditions, with each repeated 20 times for a total of 180 trials divided into 4 blocks of 45 trials. Three practice trials were completed at the start. The experiment lasted for about one hour.

## Results

Mean ratings of the protagonists’ emotional valence and intensity were calculated for each condition. Separate repeated measures ANOVA with Mask (Opaque, Transparent, and No mask) and Emotion (Positive, Neutral, and Negative) were run for each measure. Interactions were followed up with separate ANOVAs conducted for each emotion as a function of Mask. Greenhouse–Geisser corrected degrees of freedom are reported when Mauchly’s test indicated that sphericity assumptions were violated. Any follow-up paired t-tests (two-tailed) were Bonferroni corrected. Nonsignificant and marginal results were further contextualized with Bayes factor (BF_01_) analyses, which were calculated using JASP and assumed prior of no difference between means.

### Emotional valence

**Fig. 2:**
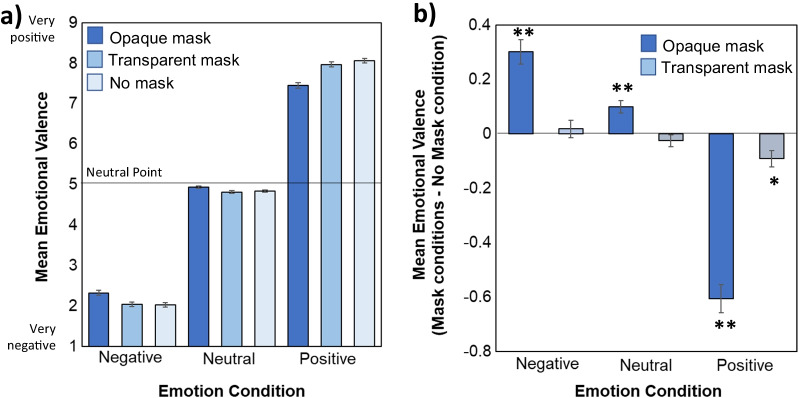
**a** Assumed emotional valence of protagonists during each Emotion and Mask condition on a scale from 1/very negative to 9/very positive. **b** Comparison of valence ratings for protagonists wearing opaque and transparent masks relative to those with no masks as a baseline for each Emotion condition (calculated via subtracting the no mask baseline from the mask conditions). Larger magnitude bars indicate a larger difference relative to the no mask condition, with * and ** indicating a significant difference from the No mask condition at *p* < .05 and *p* < .01, respectively. Error bars represent standard error of the mean

Figure 2 illustrates mean emotional valence ratings as function of Emotion and Mask type. Validating our main manipulation, a main effect of Emotion (*F*(1.28, 155.94) = 2966.38, *MSE* = 1.58, *p* < 0.001, *ηp*^*2*^ = 0.96; Fig. 2a) indicated that protagonists depicted in the negative emotion condition were assumed to have more negative emotional valence than those depicted in the neutral and positive conditions. Further, those depicted in the positive condition were assumed to have more positive valence than those shown in the neutral condition (all *ps* < 0.001).

We reasoned that opaque masks would have a detrimental effect on inferences about the protagonist’s emotional states and transparent masks have a potential mitigating effect. This prediction was supported by an interaction between Mask and Emotion (*F*(2.31, 281.25) = 84.81, *MSE* = 0.17, *p* < 0.001, *ηp*^*2*^ = 0.41; Figs. 2a, b), which qualified a significant main effect of Mask (*F*(1.88, 229.42) = 7.94, *MSE* = 0.058, *p* = 0.001, *ηp*^2^ = 0.061). Additional repeated measures ANOVAs conducted on each emotion condition showed that there were significant main effects of Mask in the positive (*F*(1.53, 186.15) = 102.49, *MSE* = 0.17, *p* < 0.001, *ηp*^*2*^ = 0.46), neutral (*F*(2, 244) = 17.62, *MSE* = 0.030, *p* < 0.001, *ηp*^*2*^ = 0.13), and negative conditions (*F*(1.78, 216.94) = 37.58, *MSE* = 0.11, *p* < 0.001*, ηp*^*2*^ = 0.24).

The negative impact of opaque masks was larger in the positive emotion condition relative to both the negative and neutral conditions (*ps* < 0.001) and larger in the negative emotion condition than in the neutral condition (*p* < 0.001).^5^ Figure 2b demonstrates the impact of mask types on valence ratings, relative to no mask, which represents typical social conditions. In the positive emotion condition, participants rated the protagonists wearing opaque (*p* < 0.001) and transparent masks (*p* = 0.010) as feeling less positive than those wearing no masks. Participants also rated the protagonists wearing opaque masks as feeling significantly less positive than those with transparent masks (*p* < 0.001). This shows that ratings for individuals wearing clear masks were significantly closer to the ratings for individuals wearing no masks. In the neutral emotion condition, participants rated protagonists wearing opaque masks as feeling more positive (note that this is closer to the neutral scale point of 5) than those with no masks (*p* < 0.001). Once again, this effect was eliminated with clear masks as there were no differences between transparent and no mask conditions (*p* = 0.76, *BF*_*01*_ = 5.26). Finally, in the negative emotion condition, participants’ emotional valence ratings for protagonists wearing opaque masks were less negative than for protagonists with no masks (*p* < 0.001). This negative occlusion effect was also eliminated when participants wore transparent masks with no difference in ratings between transparent and no mask conditions (*p* = 0.1, *BF*_*01*_ = 8.54).

To summarize, wearing opaque masks resulted in assumptions of the individual feeling more neutral in each emotion condition, but transparent masks either significantly reduced (positive condition) or eliminated (neutral and negative conditions) this impact.

### Emotional Intensity

**Fig. 3:**
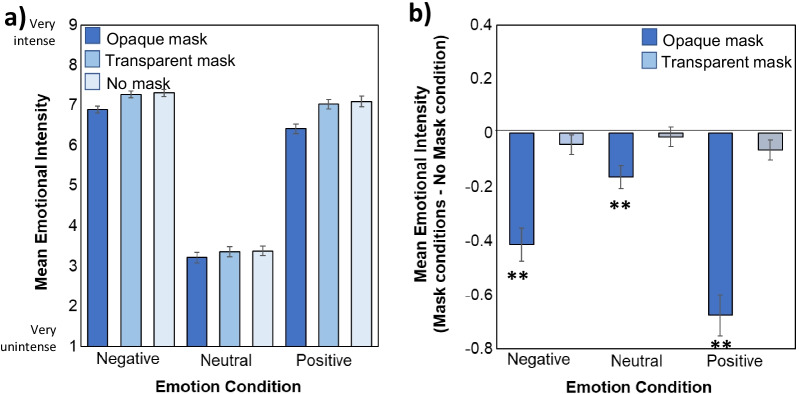
**a** The assumed emotional intensity of protagonists during each Emotion and Mask condition on a scale from 1/very un-intense to 9/very intense. **b** Comparison of intensity ratings for protagonists wearing opaque and transparent masks relative to those with no masks for each Emotion condition (calculated by subtracting the no mask baseline from the mask conditions). Bigger bars indicate a bigger difference from the No mask condition, with * and ** indicating a significant difference from the No mask condition at *p* < .05 and *p* < .01, respectively. Error bars represent standard error of the mean

Analyses conducted on emotional intensity paralleled those conducted on emotional valence. Figure 3 illustrates mean emotional intensity ratings as function of Emotion and Mask type. A main effect of Emotion (*F*(1.71, 208.48) = 501.35, *MSE* = 3.90, *p* < 0.001, *ηp*^*2*^ = 0.80; Fig. 3a) indicated that protagonists depicted in the negative emotion condition were rated as having higher emotional intensity than those depicted in the neutral (*p* < 0.001) and positive (*p* = 0.018) emotional conditions. Protagonists depicted in the positive emotional condition were rated as having higher emotional intensity than those depicted in the neutral emotional context (*p* < 0.001).

There was also a main effect of Mask (*F*(1.41, 172.33) = 83.16, *MSE* = 0.33, *p* < 0.001, *ηp*^*2*^ = 0.41; Fig. 3a) which was once again qualified by an interaction between Mask and Emotion (*F*(2.86, 349.00) = 18.20, *MSE* = 0.19, *p* < 0.001, *ηp*^*2*^ = 0.13). There was a reliable effect of mask type on intensity ratings in each positive (*F*(1.40, 170.68) = 70.28, *MSE* = 0.35, *p* < 0.001, *ηp*^2^ = 0.37), neutral (*F*(1.85, 225.36) = 10.85, *MSE* = 0.10, p < 0.001, *ηp*^*2*^ = 0.082), and negative emotional condition (*F*(1.54, 187.87) = 37.73, *MSE* = 0.22, *p* < 0.001, *ηp*^*2*^ = 0.24),

The effect of opaque masks was larger in the positive condition than both the negative and neutral conditions (*ps* < 0.001) and larger in the negative condition than in the neutral condition (*p* = 0.001). Figure 3b shows this result whereby protagonists wearing opaque masks in each emotion condition were rated to have less emotional intensity than those wearing no masks (*ps* < 0.001). Importantly protagonists with transparent masks were not perceived differently than those with no masks (1 > *ps* > 0.32, 2.78 < *BF*_*01*_ < 9.35) once again providing evidence for the mitigating effect of clear masks on affective theory of mind. In summary, while wearing opaque masks resulted in ratings of less intense emotion in protagonists, this emotional blunting effect was not present when protagonists wore transparent masks.

## Discussion

The results of Experiment 1 indicated that inferring emotional states of individuals wearing opaque masks was altered relative to inferring emotional states of individuals wearing transparent masks or no masks. Thus, in line with our predictions and prior evidence (e.g., McCrackin et al., 2022; Carbon, 2020; Grundmann, Epstude & Scheibe, 2020; Noyes et al., 2021), wearing opaque masks impairs affective theory of mind such that faces covered by opaque masks are interpreted as having emotional valence and intensity closer to neutral than faces wearing no masks or clear masks.

In Experiment 2 we investigated whether the ability to share an inferred emotional state with the protagonist, or their affective empathy, would similarly be impacted by face occlusion.

## Experiment 2

In Experiment 1, we demonstrated that wearing opaque masks altered participants’ ability to infer the mask wearer’s emotional state. Wearing transparent or clear masks ameliorated this negative effect. In Experiment 2, we investigated whether wearing masks also impacted the ability to *share* this interpreted emotional state with the protagonist. This, so-called affective empathy, (Decety, Lewis & Cowell, 2015; de Vignemont & Singer, 2006; Lieberman, 2007; Kanske et al., 2015) involves emotional “catching” (Decety & Hodges, 2006) or “contagion” (Hatfield et al., 2011) that can be both positive and negative in valence (see Morelli, Lieberman & Zaki 2015, for a review), such as feeling happy when a friend is laughing or feeling upset when they are crying. Affective empathy is an important facet of socioemotional communication and has been associated with increased social competence (Allemand et al., 2015) and prosociality (Telle & Pfister, 2016).

There is good reason to believe that affective empathy is altered by face occlusion. Affective empathy originates from another individual’s emotions, and, as such, recognizing their emotional expressions accurately (Besel & Yuille, 2010) and making complex inferences about their emotional states (Goubert et al., 2005) are often needed for an emphatic response (Clark, Winkielman, & McIntosh, 2008). Experiment 1 data as well as prior work (e.g., McCrackin et al., 2022; Carbon, 2020; Grundmann, Epstude & Scheibe, 2020; Noyes et al., 2021) has shown that both basic emotion recognition and affective theory of mind judgments are impacted by face occlusion. Thus, it is reasonable to hypothesize that wearing face masks may also impact the degree to which we are able to share those emotions with others.

To investigate this question, in Experiment 2 we used the same general procedure as in Experiment 1, with protagonists depicted in positive, neutral, and negative emotional contexts wearing opaque, transparent, or no masks. Here we asked participants to rate how much empathy they felt for each protagonist and the valence of that shared emotion. We predicted that face occlusion would be linked with lowered affective empathy (i.e., lower empathy ratings and shared emotional valence ratings closer to neutral). Based on the findings from Experiment 1, we also reasoned that the ratings of empathy for protagonists wearing transparent masks should be comparable to empathy ratings for protagonists wearing no masks.

### Participants, apparatus, stimuli, design, and procedure

One hundred and twenty-seven new participants were recruited through Prolific Academic (41 male, 83 female, 1 other, 2 unknown, mean age: 31.68, *SD* = 0.95, mean years of education from grade one: 15.79, *SD* = 0.25).^6^

Apparatus, stimuli, design, and procedures were identical to Experiment 1 except that participants were asked to rate *(a)* how much empathy they felt for the protagonist and *(b)* the valence of the emotion they were sharing with the protagonist. Mean empathy and emotional valence were analyzed using the same analysis procedures as in Experiment 1.

## Results

### Empathy

**Fig. 4:**
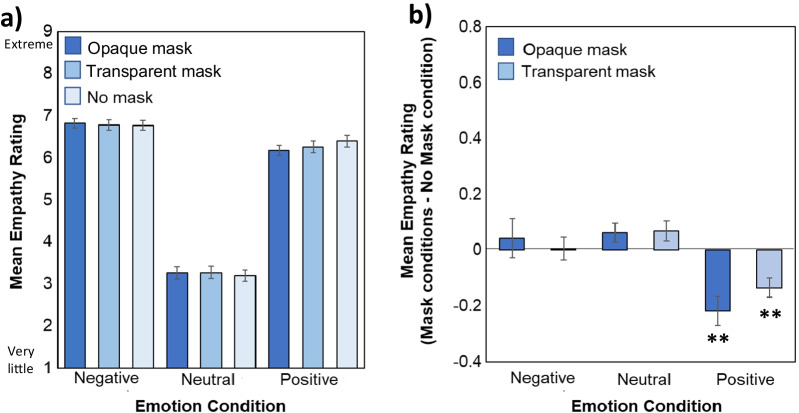
**a** Ratings of empathy for the protagonist during each Emotion and Mask condition on a scale from 1/very little to 9/extreme. **b** Comparison of empathy ratings for protagonists wearing opaque and transparent masks relative to those with no masks for each Emotion condition (calculated by subtracting the no mask condition from each mask condition). Bigger bars indicate a bigger difference from the No mask condition, with ** indicating a significant difference from the no mask condition at *p* < .01. Error bars represent standard error of the mean

Figure 4 illustrates mean empathy ratings as a function of Emotion and Mask type. A main effect of Emotion (*F*(1.67, 210.01) = 357.02, *MSE* = 4.72, *p* < 0.001, *ηp*^*2*^ = 0.74; Fig. 2a) indicated that more empathy was felt for protagonists presented in the negative and positive conditions relative to those presented in the neutral condition, and for those in the negative condition more than those in the positive condition (all *ps* < 0.001).

While the main effect of Mask (*F*(1.59, 200.77) = 0.71, *MSE* = 0.25, *p* = 0.49, *ηp*^*2*^ = 0.006) was not reliable, there was a Mask x Emotion interaction (*F*(3.44, 433.30) = 7.59, *MSE* = 0.13, *p* < 0.001, *ηp*^*2*^ = 0.057; Fig. 2a) which qualified this result (e.g., Loftus, 1978). Separate ANOVAs conduced on each emotion condition indicated that mask type had no impact on empathy experienced during negative (*F*(1.51, 190.02) = 0.32, *MSE* = 0.27, *p* = 0.66, *ηp*^*2*^ = 0.003, *BF*_*01*_ opaque vs. no mask = 8.53) or neutral (*F*(2, 252) = 2.35, *MSE* = 0.080, *p* = 0.10*, ηp*^*2*^ = 0.018; *BF*_*01*_ opaque vs. no mask = 1.93) conditions, but it did affect ratings in the positive emotion condition (*F*(1.67, 210.39) = 12.02, *MSE* = 0.15, *p* < 0.001, *ηp*^*2*^ = 0.09). Mean empathy ratings for protagonists wearing opaque and transparent masks relative to no mask conditions are depicted in Fig. 4b. As seen there, participants reported feeling significantly less positive empathy for individuals who wore opaque (*p* < 0.001) and transparent (*p* < 0.001) masks relative to those who wore no masks, and to a similar degree (*p* = 0.23, *BF*_*01*_ = 2.20). In summary, opaque masks resulted in the experience of less positive empathy, and transparent masks did not appear to reduce the impact.

### Valence

**Fig. 5:**
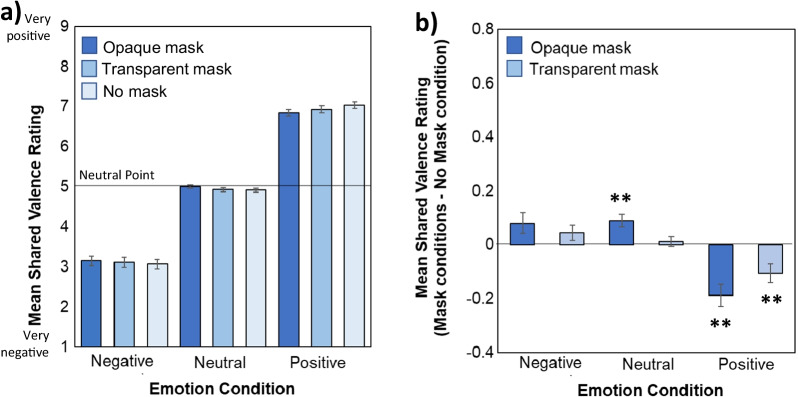
**a** Ratings of emotional valence shared with the protagonist during each Emotion and Mask condition on a scale from 1/very negative to 9/very positive. **b** Comparison of shared valence ratings for protagonists wearing opaque and transparent masks relative to those with no masks for each Emotion condition (calculated by subtracting the no mask from each mask condition). Larger bar magnitude indicates a larger difference from the No mask condition, with ** indicating a significant difference from the no mask baseline at *p* < .01. Error bars represent standard error of the mean

Figure 5 shows mean emotional valence ratings as a function of Emotion and Mask type. In line with the empathy data, a main effect of Emotion (*F*(1.18, 149.08) = 439.93, *MSE* = 5.35, *p* < 0.001, *ηp*^*2*^ = 0.78) indicated that participants felt more positive shared valence during the positive emotion conditions relative to neutral and negative emotion conditions and during the neutral relative to the negative emotion condition (all *ps* < 0.001).

Once again, there was no main effect of Mask (*F*(2, 252) = 0.49, *MSE* = 0.056, *p* = 0.49, *ηp*^*2*^ = 0.004), but a significant Emotion x Mask interaction (*F*(2.93, 368.71) = 11.49, *MSE* = 0.095, *p* < 0.001, *ηp*^*2*^ = 0.07; Fig. 2b). Separate ANOVAs conducted on each emotion condition indicated a significant main effect of Mask during both positive (*F*(2, 252) = 11.90, *MSE* = 0.094, *p* < 0.001, *ηp*^*2*^ = 0.086) and neutral emotion conditions (*F*(1.82, 228.80) = 11.65, *MSE* = 0.03, *p* < 0.001, *ηp*^*2*^ = 0.085) and a nonsignificant effect in the negative emotion condition (*F*(1.83,231.05) = 2.82, MSE = 0.08, *p* = 0.066*, ηp*^*2*^ = 0.022).

The impact of opaque masks was stronger for positive emotions than neutral ones (*p* = 0.025). The differences in valence ratings for both mask conditions relative to the no mask condition for each emotion is depicted in Fig. 4b. As depicted, opaque and transparent masks similarly reduced emotional contagion, with participants reporting in the positive condition that, relative to protagonists wearing no masks, they were sharing less positive emotion with protagonists wearing both opaque (*p* < 0.001) and transparent masks (*p* = 0.008) which did not differ (*p* = 0.118; *BF*_*01*_ = 1.26). Similarly, in the neutral emotion condition, participants reported sharing more positive valence (bringing them closer to the neutral rating of 5) for protagonists wearing opaque masks relative to those wearing no masks (*p* < 0.001). In this neutral condition, transparent masks did not produce shared valence ratings that differed from no mask condition (*p* = 1.0, *BF*_*01*_ = 8.63). In summary, opaque masks resulted in shared valence ratings closer to neutral indicating that less emotional contagion had occurred. Wearing transparent masks reduced this negative effect of occlusion for neutral, but not for positive emotions.

## Discussion

To summarize, in Experiment 2 we found that wearing both opaque and transparent face masks reduced the experience of empathy for individuals depicted in positive emotional conditions. These masks were associated with significantly reduced empathy ratings and less positive shared valence ratings in the positive emotion condition.

Interestingly, we found that wearing face masks impacted positive empathy (or sharing of positive emotional states) rather than negative empathy (or sharing of negative emotional states). One possible explanation for this result reflects the difference between the magnitudes of positive versus negative empathy. As is typical in empathy research (e.g., McCrackin & Itier, 2021a, 2021b), negative empathy was larger in magnitude than positive empathy and thus may be associated with less reliance on visual cues like facial expressions. However, given that in Experiment 1 positive affective theory of mind was also more impacted by face masks than negative affective theory of mind, it seems more likely that face masks impact inferring and sharing positive emotions more because they occlude lower face features important for perceiving happiness. Although negative empathy has traditionally received the most attention in the literature (Morelli, Lieberman, & Zaki, (2015), both positive and negative empathy has been associated with increased social competence (Allemand et al., 2015) and prosociality (Telle & Pfister, 2016). While negative empathy might facilitate reaching out to help another individual in need (Andreychik & Lewis, 2017), sharing positive emotions is thought to also be an incredibly important part of relationships (Gable, Reis, Impett, and Asher, 2018), and some have argued that it can help make relationships intrinsically rewarding by easily increasing positive affect (Duan, 2000; Telle & Pfister, 2016), even helping to offset the emotional cost of sharing negative emotions (Andreychik, 2019).

## General discussion

The 2020 worldwide Covid-19 pandemic saw a surge in the general public covering the lower face, as widespread mask adoption was implemented to prevent virus contagion (Eikenberry et al., 2020; Leung et al., 2020; Prather et al., 2020). Since facial features convey a number of important visual cues needed for successful interactions, the effects of widespread mask use for human social communication remain relatively unknown (Mheidly, 2020). Here we addressed how occlusion of the lower face features by masks impacted making inferences about another person’s emotions (i.e., affective theory of mind) and sharing of their emotional states (i.e., affective empathy). We examined these variables when protagonists wore opaque, transparent, and no masks.

In two experiments, we manipulated the type of mask (opaque, transparent, no mask) and the facial emotion which was displayed within congruent affective context (positive, neutral, and negative). In Experiment 1, we measured participants ratings of protagonists’ emotional states and their emotional valence. The results indicated that participants were generally able to infer protagonists’ emotional states, but that performance was modulated by the mask type. Specifically, opaque, but not transparent masks, altered the affective theory of mind judgements such that protagonists wearing opaque masks were rated as less emotional, including rating of more neutral emotional valence and less emotional intensity. This impact was strongest for protagonists experiencing positive emotions but was also present for those experiencing negative and neutral emotions. In Experiment 2, using the same stimuli and design, we measured how facial occlusion by masks impacted affective empathy, or the ability to share emotions with another individual. Unlike Experiment 1, the data from Experiment 2 showed that wearing both opaque and transparent face masks altered empathy ratings for the protagonists, resulting in less experienced positive empathy and more neutral shared emotional valence. Again, the effect of masks on empathy was strongest for positive emotions. In summary, the data from the two experiments converged to show that lower face coverings impacted socioemotional processing and resulting nonverbal social communication, particularly for positive emotions. The data diverged in that covering the face with transparent instead of opaque masks alleviated the impact of facial visual occlusion for affective theory of mind, but not for affective empathy. We raise and discuss four points related to these findings.

First, extending past work, our results build on the literature showing the impact of face masks on emotion recognition (McCrackin et al., 2022; Carbon, 2020; Grundmann, Epstude & Scheibe, 2020, Noyes et al., 2021) demonstrating that occluding face parts also leads to impairments in downstream socioemotional processes that depend on emotion recognition, such as those invoked by affective theory of mind and affective empathy. It is likely that the impairments in both basic emotion recognition as well as more sophisticated representations of others’ emotions stem from the visual occlusion of facial cues needed for accurate judgements of emotional expressions (McCrackin et al., 2022; Carbon, 2020; Grundmann, Epstude & Scheibe, 2020; Noyes et al., 2021). Accordingly, it is suggested that the larger impact of face masks on positive affective theory of mind and empathy was driven by covering the smile, a key diagnostic feature for the expression of happiness (e.g., Ekman et al, 1990, 1999). Indeed, facial expression recognition is considered a key component for both affective theory of mind (Baron-Cohen & Cross, 1992; Clark et al., 2008; Mier et al., 2010) and affective empathy (e.g., Clark et al., 2008; Goubert et al., 2005; Besel & Yuille, 2010), given that accurate perception of emotional expressions precedes interpretation of another person’s emotional state, which in turn impacts emotional contagion. Mirror theories of empathy also suggest that spontaneous mimicry of emotional expressions facilitate emotional contagion (Gallese, 2013; see Bekkali et al., 2021 for a review) with mimicry processes linked to increased empathy (e.g., Andréasson, 2010; Dimberg & Thunberg, 2012; Rymarczyk et al., 2016). This mimicry process is likely also impaired by masks (Kastendieck et al. 2022) which prevent perceiving emotional face features.

Second, it is important to note that while previous work has shown an impact of face masks on basic emotion recognition (e.g. McCrackin et al., 2022; Carbon, 2020; Grundmann, Epstude & Scheibe, 2020; Noyes et al., 2021), we now show that face masks impact emotional inferences and empathy even when a clear emotional context is provided, which more closely mimics everyday life (e.g., a smile might be viewed within the context of a joke). These findings likely reflect an incorporation of both facial emotion cues and emotional context, given previous work with similar paradigms that has demonstrated that other facial cues, like the gaze direction of neutral faces, interact with emotional context to impact affective theory of mind (McCrackin & Itier, 2021a) and empathy (McCrackin & Itier, 2021b) judgements. The relative importance of facial emotion (e.g., emotion from facial expressions) versus contextual information (i.e., context sentences) to affective theory of mind and affective empathy, however, remains to be investigated, with future studies examining how each of these facets affects socioemotional communication under typical and face occlusion conditions.

Third, the results from Experiment 1 indicated that one way of mitigating socio-communicative issues brought about by opaque masks would be to use transparent masks, which allow for the visual transmission of lower face cues. Our data indicated that transparent masks have the potential to restore some, but not all, aspects of socioemotional communication. Affective theory of mind judgements for protagonists wearing transparent face masks was mostly on par with affective theory of mind judgements for protagonists wearing no masks. However, similar results were not found in Experiment 2, where transparent face masks had a similar negative impact on positive empathy ratings as opaque masks. This was a surprising finding, and future research is needed to determine why transparent face masks would impact positive empathy but not affective theory of mind. One possibility is that although transparent masks allow for the transmission of visual cues, they may serve as a distraction away from key facial features involved in perceiving emotions. Another possibility is that having a barrier, even a transparent one, between the observer and the mask wearer creates a perception of social and/or emotional distance. This may be akin to creating psychological distance, which has been shown to impact affective responses (Williams et al., 2014) including dispositional empathy (Paniculangara & He, 2012). Future work is needed to provide additional information about these questions by, for example, measuring attentional allocation to face parts for faces wearing opaque, transparent, and no masks and/or assessing the psychological feeling of closeness between the observer and the protagonist.

Finally, our results provide new insights into the importance of face perception in socioemotional processing beyond the specific Covid-19 pandemic context. While there is a large amount of research investigating the impact of face feature perception on social processes (Hugenberg & Wilson, 2013 for review), there has been little work with test procedures that better approximate real-world scenarios which often utilize both facial cues and emotional context. Similar paradigms can be used to investigate how perception of face features interacts with other types of information, such as auditory cues or social context like person familiarity, and to understand how perceiving upper face features as opposed to the lower face features may affect socioemotional processes (e.g., Noyes et al., 2021). Eye perception is especially featured in prominent social cognition theories (e.g., see Itier & Batty, 2009; Cañigueral & Hamilton 2019; George & Conty, 2008 for reviews), and so paradigms combining affective context with eye occlusion could be very informative.

We note that the effects of face masks were relatively small in magnitude, so the real-world impact of these effects warrants further investigations. However, we can speculate that as participant ratings on the scales ranged on average from around 2–8 in Experiment 1 and 3–7 in Experiment 2, the mask occlusion has resulted in shifts in ratings of around 3.5–10% depending on the condition. Beyond theoretical conceptualization, our results also touch on communication in settings which normatively use face coverings, such as hospitals, and educational settings which have seen an increased emphasis on mask wearing. The perception (e.g., Clark et al., 2008; Goubert et al., 2005; Besel & Yuille, 2010) and mimicry (e.g., Andréasson, 2010; Dimberg & Thunberg, 2012; Rymarczyk et al., 2016) of facial expressions is thought to be the key for engaging in affective theory of mind and experiencing affective empathy in these contexts (e.g., Allemand et al., 2015; Telle & Pfister, 2016; e.g., Decety & Fotopoulou, 2015). In clinical situations, increased therapist empathy and emotion understanding is linked to better client outcomes (Elliott et al., 2018), and in schools, increased student empathy predicts better social and academic performance (Eisenberg, & Spinrad, 2014). In medical contexts, physician empathy is linked with increased patient compliance (Halpern, 2003) and favorable health outcomes (see Decety & Fotopoulou, 2015 for a review, Finset and Mjaaland, 2009). Beyond beneficial effects of empathy for patients, positive empathy is also important for health care providers, as it is thought to reduce burnout by offsetting the emotional cost of feeling negative empathy (Andreychik, 2019; Dyrbye et al., 2010). Our findings indicating that transparent masks restore the ability to infer emotional states from others would suggest that the adoption of transparent masks would be beneficial for improving social communication in educational and healthcare settings. The effect of transparent masks in educational settings, in particular, needs further study as removing visual input by opaque masks during critical phases of development may be associated with long lasting consequences on a range of cognitive and social abilities.

In sum, our study demonstrates that wearing opaque face masks impairs human social communication and that wearing transparent masks provides a partial mitigation of this impairment. Future studies are needed to understand the full impact of face occlusion for human social communication, across a wide range of educational, developmental, and professional settings.

## Data Availability

The study was pre-registered (https://osf.io/8pa9u). Anonymized raw/summarized data are available at https://osf.io/t698c/?view_only=dacda69474074b10820ee5c1ce47aa0d.

## Appendix 1

List of emotional sentences used to provide emotional context. Male and female variations, as well as positive, negative and neutral variations are indicated.

**Table:**

1. His/her pet dog was saved/fed/killed yesterday afternoon
2. His/her pet cat was saved/fed/killed yesterday afternoon
3. He/she loves/does/hates the job and the boss that he(she) works with
4. His/her partner’s life was saved/partner went shopping/partner’s life was lost yesterday morning
5. His/her son’s life was saved/son was delayed behind/son’s life was lost after a bad car crash
6. He/she was just reunited/doing housework with/separated from his(her) partner
7. His/her child was reunited with/at his(her) workplace with/separated from him(her) today
8. He/she aced his(her)/marked the/failed his(her) very important physics test
9. He/she aced his(her)/marked the/failed his(her) very difficult math exam
10. He/she knows his partner is so in love/not shopping/not in love with him(her)
11. He/she was accepted/also there/rejected at the job interview
12. He/she got accepted by/to read about/rejected by the school he wanted
13. His/her partner told him(her) she(he) really does love him(her)/really does love cats/no longer loves him(her)
14. He/she knows right now that his(her) partner is faithful/shopping/cheating
15. His/her partner has decided to marry/drive with/divorce him(her)
16. His/her cat’s life was saved/toy was bought/life was lost yesterday afternoon
17. His/her pet dog was found/fed/lost yesterday afternoon
18. His/her close childhood friend just passed by/passed the store/passed away today
19. He/she found an organ match to save/studied organ matches with/found no organ match to save his(her) sister
20. His/her newborn was saved/fed/killed yesterday afternoon

## Footnotes

1 Data from one hundred and twenty-four (124) participants were collected. One participant was removed from the final analysis based on preregistered exclusion criteria of overall performance ± 2.5 SD away (score of 17) from the sample mean (*M* = 47.04, *SD* = 8.03) on the Toronto Empathy Questionnaire and having more than 20% of experimental trials lost due to errors (i.e., anticipatory responses given in less than 500 ms) or no answers. Data quality was overall high, with an average of 179.90/180 (SD = 0.32) arousal responses and 179.87/180 (SD = 0.42) valence responses completed. All participants were fluent in English, possessed normal or corrected-to-normal vision, had no ongoing diagnosis of a mental health condition, no mild cognitive impairment or dementia, no history of head trauma resulting in loss of consciousness, and a minimum 95% Prolific approval rate.

2 FACES Identities: Male—008_y_m, 013_y_m, 016_y_m, 025_y_m, 037_y_m, 049_y_m, 057_y_m, 062_y_m, 066_y_m, 072_y_m, 081_y_m, 089_y_m, 099_y_m, 105_y_m, 109_y_m, 114_y_m, 119_y_m, 123_y_m, 127_y_m, 135_y_m, 144_y_m, 147_y_m, 153_y_m, 160_y_m, 170_y_m, 175_y_m. Female- 010_y_f, 022_y_f, 028_y_f, 034_y_f, 040_y_f, 048_y_f, 054_y_f, 063_y_f, 069_y_f, 071_y_f, 085_y_f, 090_y_f, 098_y_f, 101_y_f, 106_y_f, 115_y_f, 125_y_f, 132_y_f, 134_y_f, 140_y_f, 162_y_f, 163_y_f, 171_y_f, 173_y_f, 177_y_f, 182_y_f.

3 KDEF Identities: Male—M08, M11, M31, M35; Female—F05, F13, F19, F26.

4 After the computerized task, participants completed the Toronto Empathy Questionnaire (TEQ; REF) and the Autism Spectrum Quotient (AQ; Baron-Cohen et al., 2001). The TEQ assesses affective empathy, defined as sharing someone’s emotional state, and contains 16 questions that capture facets of this construct (i.e., emotional contagion, emotion comprehension, sympathetic physiological arousal, and conspecific altruism; Spreng et al., 2009). Responses are made on a five-point Likert scale ranging from 1 (never) to 5 (always), with higher scores indicating higher trait empathy. Scores on the TEQ were used to ensure that participants self-reported experiencing typical ranges of trait empathy. The AQ was completed to assess social competence for an ongoing project (not reported or analyzed for this study).

5 Note that the effects of opaque masks are small in magnitude. We examined our data and found that there were no participants who either consistently gave a valence rating above 0 for negative trials (incorrect assumption of positive valence) or below 0 for positive trials (incorrect assumption of negative valence) when faces were masked. Thus, masks changed the degree of emotionality inferred, but did not result in complete reversals of judgements.

6 128 responses were collected. Data from one participant were removed for both high data loss and TEQ score (score of 8; group *M* = 46.14, *SD* = 7.39). Data quality was high once again, with participants completing an average of 179.90/180 (SD = 0.32) empathy and 179.87/180 (SD = 0.42) shared valence responses. Note that two participants did not provide demographic information.
