# Correction: Transparent masks reduce the negative impact of opaque masks on understanding emotional states but not on sharing them

**DOI:** 10.1186/s41235-022-00427-0

**Published:** 2022-08-01

**Authors:** Sarah D. McCrackin, Sabrina Provencher, Ethan Mendell, Jelena Ristic

**Affiliations:** Department of Psychology, McGill University, 2001 McGill College Avenue, Montreal, QC H3A 1G1 Canada

## Correction to: Cognitive Research: Principles and Implications (2022) 7:59 https://doi.org/10.1186/s41235-022-00411-8

The original article (McCrackin et al. 2022) contained an error in attribution of equal contribution in the authorship. This has since been corrected to show Sabrina Provencher and Ethan Mendell as the correct equal contributors.
